# Barriers and solutions for introducing donation after circulatory death (DCD) in Japan

**DOI:** 10.1007/s10047-024-01491-7

**Published:** 2025-01-15

**Authors:** Yasuhiro Kotani

**Affiliations:** Department of Cardiovascular Surgery, Faculty of Medicine, Dentistry and Pharmaceutical Sciences, Okayama University and, Okayama University Hospital, 2-5-1 Shikata-cho, Kita-ku, Okayama City, Okayama Prefecture 700-8558 Japan

**Keywords:** Heart transplanatation, Donation after circulatory death, Machine perfusion

## Introduction

Since the revision of Act on Organ Transplantation (Organ Transplant Act) in 2010, the number of heart transplantation from donation after brain death (DBD) has been steadily increasing, and in 2023, there were 115 transplants, exceeding 100 cases per year for the first time [1]. This is the result of the tireless efforts of organ donor facilities, the Japan Organ Transplant Network, and organ transplant centers, as well as the generosity of donors and their families. However, while the number of DBD heart transplantation is increasing, the number of people on the waiting list for heart transplantation is 861 as of January 31, 2024, and the average waiting period is more than 4 years, showing no improvement at all [2]. One of the reasons for this is the excellent management of heart failure in Japan, including the use of left-ventricular assist system. Problem in Japan is that the number of left-ventricular assist system implantation does not match the number of transplantation. Therefore, increasing the number of donors is an urgent issue in the current situation of heart transplantation in Japan. In contrast, heart transplantation of donation after circulatory death (DCD) has been increasing over the past decade and has shown excellent results in the other countries [3]. In this paper, the actual situation and problems of heart transplantation from DCD in other countries are summarized, and the problems and solutions to implicate DCD heart transplantation in Japan are also discussed.

## Current status of DCD heart transplantation overseas

### Clinical results

In Australia, where DCD heart transplantation began in 2014, the 10-year survival rate was reported to be 72%, which was not different from that of DBD (62%) [4]. In the United States, where the percentage of DCD heart transplantation is rapidly increasing to approximately 14% of all heart transplants in recent years, the 1-year survival rate after DCD heart transplantation was reported to be 94.3%, which was not inferior to that of DBD (92.4%) [5]. These results indicate that with appropriate donor selection and methods of preservation and perfusion (transport) of the explanted heart, the results are comparable to those of DBD, resulting the increased use of DCD worldwide. Also, this is expected to lead to further increases in Western countries in the future [6]. The percentage of DCD in all heart transplants varies from country to country, but in the aforementioned Australia, it was 26.9%, and in the United States, it was 14.1%, while in Belgium, it was 43% and in the Netherlands, where the use of DCD started in 2021, it accounted for 63% [7].

### Procurement method

In the retrieval of DCD heart, the heart can be reperfused with two methods: normothermic regional perfusion (NRP) by extracorporeal membrane oxygenation (ECMO) and direct procurement and perfusion (DPP). In NRP, after a hands-off period of 2–5 min is usually allowed upon a pronouncement of death, a sternotomy is made and establishment of ECMO is initiated. At this time, the cerebral blood vessels must be occluded and only organs other than the brain must be perfused. The advantages of this procedure are: (1) it maximizes preservation of cardiac function by minimizing warm ischemia from cardiac arrest to reperfusion, and (2) it makes it possible to evaluate cardiac function before the retrieval. Once the heart is retrieved, the process is the same as heart transplantation from DBD, and it is superior from a cost standpoint, because it does not necessarily require a machine perfusion. One of the ethical concern is that cerebral perfusion from collateral blood vessels may not be zero during NRP. Previous reports showed that there are many collaterals that may perfuse the brain during body perfusion by ECMO by NRP even clamping the neck vessels [8, 9]. This may trigger brain reperfusion and potential functional recovery which could be against the determination of circulatory death defined as permanent cessation of entire brain function.

DPP allows the heart alone to be transported while being perfused in a machine perfusion device. DPP has the advantage of allowing evaluation of cardiac function in a perfusion device prior to transplantation and is particularly important for heart transplantation from DCD where there is concern about the function of the transplanted heart. In Western countries, a commercial extracorporeal perfusion device called OCS™ (TransMedics, MA, USA) is widely used, but its disadvantage is that it is more expensive than NRP (NRP 28,000 USD vs. DPP 100,000 USD, per transplant, as of January 2024, USA). Who pays the cost for the use of OCS™ depends on the hospital in the United States. For instance, Brigham and Women’s hospital in Boston charges organ acquisition cost of 118,000 USD to patient’s health insurance. Total cost of OCS per transplant accounts for 150,000–200,000 USD, including 80,000 USD for disposable parts of OCS, 20,000 USD for surgeon and technician who retrieve the heart, and the cost for the transportation using the jet. Taken together, 30,000–80,000 USD are covered by the hospital.

In terms of choice of procurement methods, the use of NRP accounts for 32% in the United States and 8% in England. All procurements are done by DPP in Australia, Netherlands, and Austria. On the other hands, Spain, Belgium, and Italy only use NRP [7].

### Cardiac function evaluation

In DCD heart transplantation, there is a period of warm ischemia between the cessation of life support (primarily ventilation), cardiac arrest, pronouncement of death, and the end of hands-off. Because of the possibility of decreased cardiac function after transplantation, it is essential to assess cardiac function prior to the implantation. The method of assessing cardiac function differs depending on the method of procurement. In NRP with ECMO, cardiac function is usually assessed in vivo after 30 min of cardiac reperfusion.

On the other hand, in the case of DPP with a perfusion device, the evaluation is performed in the device, but since the heart is in a non-working state with no preload or afterload, an accurate evaluation in the same environment as in vivo is not possible. In the OCS™ described above, lactate concentration is used as a metabolic index of cardiac function, and a lactate concentration of less than 5 mmol/l is a prerequisite for implantation. The disadvantage is that this is a metabolic and not a functional assessment, and future development of techniques to assess cardiac function in a similar environment to in vivo is necessary.

### Guideline and protocol

As mentioned above, cardiac function in DCD heart transplantation depends on warm ischemic time, so the ideal is to reperfuse the heart within 30 min. To achieve this, it is essential to have a detailed protocol for the process from withdrawal of life support to reperfusion. In the England, which pioneered the use of DCD, the National Health Service produced a report on the Consensus Meeting on Organ Donation after Circulatory Death in Organ Donation and Transplantation. In particular, as national protocols for heart transplantation, detailed protocols for preparation for extraction, how to establish NRP, extraction methods, etc. are clearly described [10].

Similarly, in Australia, the government's Organ and Tissue Authority has established detailed guidelines from donor selection to removal [11]. In Austria, where DCD heart transplantation started relatively recently in 2019, clinical implementation has also begun, following detailed definition of where end-of-life care will be provided, how donors will be selected and evaluated, and how the use of organs other than the heart will be coordinated. In Austria, clinical implementation began only after detailed guidelines were established on where end-of-life care should be provided, how donors should be selected and evaluated, and how the use of organs other than the heart should be coordinated [12]. In Australia, it is permissible to disinfect and drape the heart before hands-off is completed, and heart retrieval can begin immediately. In the Netherlands, where DCD heart transplantation started relatively recently in 2021, there are strict rules for organ retrieval, such as the use of controlled DCD, hands-off for 5 min, use of OCS™ for reperfusion, and functional warm ischemia of less than 30 min from systolic blood pressure of 50 mmHg or less to injection of cardioplegic solution [7].

## Problems of DCD heart transplantation in foreign countries

### Laws

In the United States, there is a law called the Uniform Determination of Death Act (UDDA) [13] regarding the definition of death. According to this law, death is defined as (1) the irreversible cessation of cardiopulmonary functions or (2) the irreversible cessation of all brain functions including the brainstem. However, this law was enacted in 1981 and does not reflect the current situation in which DCD is used, and there have been efforts to revise it in recent years. In Europe, the European Society for Organ Transplantation consensus statement on NRP states that "permanent" means that no attempt has been made to restart circulation and there is no prospect of natural recovery, and therefore, the term "irreversible cessation" should be used. The description states that "permanent cessation" is more appropriate than "irreversible cessation", because "permanent" means that there is no prospect of natural recovery without an attempt to resume circulation. Incidentally, "permanent cessation" is defined as (1) continuous apnea for 5 min, (2) circulatory arrest, and (3) neurological unresponsiveness [14]. The United States is the only country in the world to have a legal definition of death, but even in the United States, the definition does not meet modern interpretations, and the debate over the definition of death is likely to continue.

### Ethical aspects

It has been argued that there are ethical issues with this method of postmortem reperfusion, because the brain has collateral blood channels [8] and NRP may slightly perfuse the brain. In other words, if there is activity in only one part of the brain, it does not meet the UDDA definition of death as the irreversible cessation of all brain function. In the U.S., whether or not NRP is performed is determined by hospital-specific or regional rules (there are no uniform rules in the U.S.). In any case, it must be ensured that NRP does not cause cerebral perfusion. In Austria, carotid Doppler confirmation is performed during NRP. In the United States, on the other hand, monitoring including EEG is not currently used.

### Frequency of use of extracted hearts

In DBD heart transplantation, the probability of transplantation of an explanted donor heart is more than 95%. On the other hand, in DCD heart transplantation, the rate is about 60–80% according to reports from other countries [7]. Reasons for this include technical problems, malignancy of other organs, size mismatch, and poor cardiac function. In other cases, cardiac arrest may not occur even after life support is withdrawn. In these cases, in addition to the labor and travel costs of the harvesting team, the costs of the cardiopulmonary bypass circuit and ECMO are required, but the heart cannot be transplanted.

### Cost

For example, in the U.S., DPP by OCS™ is expensive at 100,000 USD, and although ethical issues remain, many hospitals still use NRP (28,000 USD) with ECMO [15]. In the U.S., there is also a contract company that performs organ procurement and will dispatch and transport a harvesting team for 100,000 USD per harvest [16]. On the other hand, if the harvest is performed by a team from a transplant facility, the cost is as low as USD 28,000. In addition, while the cost of a surgeon is $5000 per visit, the cost of a physician assistant is $300, and some facilities employ highly trained physician assistants as part of the transplant team. As noted above, the likelihood of heart failure is higher with DCD than with DBD, and many centers prefer the less expensive procedure (since the transplant facility pays for the harvest).

## Barriers and solutions for DCD heart transplantation in Japan

### Laws

Although there is no law defining death in Japan, the Organ Transplant Act (1997) contains a definition of organ transplantation, stating that "organs for use in transplantation procedures may be removed from a dead body (including the body of a brain-dead person)." [17] Since dead bodies include those from cardiac death, it can be interpreted that organs can be removed from cardiac death donors. In fact, transplants of eyes, kidneys, and pancreases have been performed from DCD. The definition of death in Japan is the three signs of death: (1) cessation of spontaneous breathing, (2) cessation of heartbeat, and (3) pupil dilation (loss of reflexes). Although these definitions are clinically consistent with the definitions of death in other countries, we believe that the law should be amended to specify these definitions to promote transplantation from DCD in the future.

### Withdrawal of life support therapy

To perform heart transplantation from a controlled DCD, it is necessary to withdraw life support, but there have been ethical issues in Japan until now. The Guidelines for End-of-Life Care in Emergency and Intensive Care Medicine (Japanese Society of Intensive Care Medicine, Japanese Association for Acute Medicine, Japanese Circulation Society) [18] published in November 2014 defines the end of life as "the time when it is judged that there is no hope of survival even with appropriate treatment" for acutely ill patients being treated in intensive care units and other settings. It also mentions the option of withdrawing life-supporting devices, such as ventilators, pacemakers, and circulatory assist devices at the end of life when the medical team determines that the patient has irreversible total brain dysfunction or irreversible dysfunction of multiple organs necessary for life support, and there is no hope of recovery, which could be interpreted as not prohibiting the withdrawal of life-supporting devices. However, the purpose of withdrawing life support must never be to harvest organs. Withdrawal of life-supporting equipment is a decision made as part of end-of-life care for patients, and for this reason, it is essential that laws and guidelines be developed.

### Guidelines

In Japan, there are no guidelines for organ procurement in DCD. Therefore, it is important to clarify the diagnostic criteria for irreversible dysfunction of multiple organs, including the brain and heart. With regard to the declaration of death, it is necessary to specify the three signs of death and include criteria for pulse and blood pressure values as indicators of circulatory arrest. The hands-off time also needs to be clarified. To shorten the warm ischemic time, it is important to shorten the time from the end of hands-off to chest opening and cardiac reperfusion, and it is necessary to consider whether life support should be discontinued in the operating room or whether the patient should be transferred to the operating room after hands-off, in addition to the environment of the provider facility and the acceptance status of the patient's family. However, if cardiac function is compromised by prolonging normothermic ischemia time, this would be a complete reversal of the situation, and a limit on normothermic ischemia time should be established. As mentioned above, there are two methods of cardiac reperfusion, NRP and DPP, and it is important to describe and clarify each process in the guidelines. Especially in the case of NRP, there is an ethical aspect of establishing ECMO for deceased donors and performing perfusion of organs other than the brain that needs to be understood by the public. In addition to heart transplantation, comprehensive guidelines are expected for lung and abdominal organ transplantation. DCD kidney and pancreas transplantation is currently performed in Japan. For example, pretreatment, such as cannulation is allowed after diagnosis of clinical brain death to retrieve these organs. Protocols and guideline for each organ retrieval as well as entire organs to show the details of the use of DCD are necessary.

### Potential donors

In actual end-of-life care, there are cases in which brain death cannot be determined despite irreversible brain damage. These include (1) cervical cord injury, (2) severe corneal damage or loss, and (3) patients undergoing circulatory support such as ECMO. These patients are candidates for cardiac arrest donors, but end-of-life care should never be provided with organ donation as the primary goal. After providing patient-centered end-of-life care, the patient's irreversible organ dysfunction should be established by appropriate diagnosis, and organ donation should be presented to the family as one of the options. Although medical professionals involved in end-of-life care do not provide treatment for the purpose of organ donation, the number of potential DCD patients and the policies of donor facilities should be investigated in the future.

### Burden on the donation hospitals

At present, there are 914 hospitals where organs can be donated, but only 414 are willing to donate organs, and only 233 have actually done. This is due to factors such as the burden of explaining and following up with the patient’s family and the work involved in determining brain death [19]. In response to this situation, the Organ Donation Facility Coordination System Establishment Project, the In-Hospital System Improvement Support Project, and the Prefectural Support Project were introduced. For instance, experienced donor hospital can help other hospitals to identify the potential donors and donor procurement process. While these projects have made it possible to increase the number of donations, there are still problems to be solved, such as the increased burden on prefectural coordinators.

### Cost

Commercial extracorporeal perfusion systems (OCS™) used overseas cost more than 10 million yen per use, and their introduction in Japan would be practically difficult for the present. Under these circumstances, the development of a domestically produced extracorporeal perfusion device using existing ECMO is underway and awaiting a commercially available product [8]. Currently, the cost of organ donation other than kidney, pancreas, and cornea from DCD is not recognized as an insured medical treatment, and the full amount is borne by the donor facility. Negotiations with the Ministry of Health, Labor and Welfare through relevant academic societies will be necessary in the future.

## Conclusion

In addition to the revision of the Organ Transplantation Act, the efforts of donation and transplantation facilities, and the support of the national and prefectural governments, the Japan Organ Transplant Network, and organ transplant coordinators, Japan has achieved an unprecedented number of heart transplants. However, in the current situation where the number of people on the transplant waiting list is increasing, organ donation from DCD is an issue that needs to be realized. It is important to understand the data and systems of other countries that have more than 10 years of experience and incorporate them into our country, and therefore, academic societies should take the lead in developing guidelines in the future.

## Data Availability

The data that support the findings of this study are openly available.
